# Thermoset/Thermoplastic Interphases: The Role of Initiator Concentration in Polymer Interdiffusion

**DOI:** 10.3390/polym14071493

**Published:** 2022-04-06

**Authors:** Ozan Erartsın, Jamal Sayyed Monfared Zanjani, Ismet Baran

**Affiliations:** Faculty of Engineering Technology, University of Twente, 7500 AE Enschede, The Netherlands; j.seyyedmonfaredzanjani@utwente.nl

**Keywords:** thermosetting resins, thermoplastics, co-bonding, interphase, diffusion, adhesion

## Abstract

In the co-bonding of thermoset and thermoplastic polymers, the interdiffusion of the polymers results in the formation of an interphase between them. Understanding the factors influencing the interdiffusion and the resulting interphase is crucial in order to optimize the mechanical performance of the bond. Herein, for the first time, the effect of the initiator concentration of the thermoset resin-initiator mixture on the interphase thickness of co-bonded thermoset-thermoplastic polymers is investigated. The dependence of the gelation time on the initiator concentration is determined by rheometer measurements. Differential scanning calorimetry measurements are carried out to determine the speed of cure. To co-bond the polymers, pieces of already-manufactured thermoplastic plates are embedded in a resin-initiator mixture. The interphase thickness of the co-bonded polymers is measured with an optical microscope. The results of this study show that the gelation time decreases as the initiator concentration increases. This decrease leads to a significant reduction in both interphase thickness and diffusivity. For instance, increasing the initiator/resin weight ratio from 1% to 3% reduces the gelation time by 74% and the interphase thickness by 63%.

## 1. Introduction

Fiber-reinforced polymer composites (FRPC) provide not only a high strength-to-weight ratio but also exceptional properties such as high durability, stiffness, and corrosion resistance. While knowledge of the manufacturing of individual FRPC parts has reached a level of maturity, the integration and assembly of different FRPC parts is far less developed, particularly considering the co-bonding process. Co-bonding is a bonding technique in which a prefabricated part (in this case, a thermoplastic (TP) polymer) is bonded with a (neat or fiber-reinforced) thermoset polymer through a curing reaction of the thermoset resin [1,2,3,4]. The areas of application of this technique involve the bond between the pultruded profiles at the blade root, spar cap, and leading edge protection (LEP) layer and the over-infused main body of the wind turbine blade [5,6]. Although co-bonding may refer to the bonding of two parts with or without an adhesive between them [1,2,3,4,7], in this work we will focus on co-bonding without adhesives, where bonding takes place by the interdiffusion of polymers that are in contact as the curing takes place. The interdiffusion of the bonded polymers, and, subsequently, the curing of the resin result in the formation of an interphase [2,4,8,9,10,11,12]. The size and morphology of the interphase have been shown to depend on the gelation time and viscosity of the resin, the thermodynamic affinity between the polymers, and the physical state of the thermoplastic [2,4,8,9,10]. For instance, high levels of thermodynamic affinity may promote homogeneous mixing, whereas phase separation may take place at lower levels of affinity [9]. The gelation time and viscosity of the resin have competing effects on the interphase thickness [2]. Higher gelation times allow more time for the interdiffusion to take place, which eventually promotes an increase in the interphase thickness. Conversely, an increase in the resin viscosity hampers the diffusion of resin into the thermoplastic, leading to a lower interphase thickness.

It is of the utmost importance to investigate the influence of the aforementioned parameters on the TP-TS interphases. This is necessary in order to optimize the design of co-bonded hybrid composites, closing the knowledge gap in this field and promoting their more widespread use. To illustrate, for the accurate prediction of the residual stresses and the resulting process-induced deformations in the co-bonded composites, the size and the mechanical properties of the interphase are essential inputs [1,13]. Another reason why understanding the interphase is important is that the interphase morphology, which is influenced by processing conditions, plays a major role in the resulting bond strength [14]. As it is highly desirable to have stronger bonds in the joining of composites, determining the factors leading to optimum bond performance is crucial.

One way to control the cure speed of a resin-initiator mixture is to change the concentration of initiator in the mixture [15,16]. For instance, in vacuum-assisted resin transfer molding, the initiator concentration can be tuned to delay the gelation of the resin to allow sufficient time for the resin to fill the mold [15]. In the literature, studies on the effect of initiator concentration on the gelation time of neat resins are available [15,16,17,18]. Nevertheless, the effect of initiator concentration on the interphase morphology, which is crucial for the co-bonded parts, since the interphase morphology is also affected by the gelation time, has not been studied so far.

One benefit of changing the initiator concentration to tune the gelation time is that it allows one to control the gelation time without affecting other parameters controlling the interdiffusion. For instance, decreasing the temperature to increase the gelation time also reduces the viscosity of the resin, which has an adverse effect on the interphase thickness [2,19]. Tuning the initiator concentration eliminates this problem and, hence, enables one to investigate the effect of gelation time on the interdiffusion exclusively.

This study aims to investigate the effect of initiator concentration of a resin-initiator mixture on the interphase thickness of co-bonded unsaturated polyester resin (UPR) and polycarbonate (PC). UPR is a resin that is commonly used in wind turbine blades and PC is a TP polymer that could potentially be used for LEP applications. Initially, the gelation time of the resin at different initiator concentrations is measured and DSC tests are conducted to determine the cure behavior (cure speed and degree of cure). Resin-initiator mixtures with different initiator concentrations are co-bonded to PC and the interphase thickness is measured using optical microscopy. To investigate the diffusion kinetics, the diffusivity of the mixtures is calculated based on Fick’s second law of diffusion using the measured interphase thicknesses and gelation times. Finally, the measured interphase thickness and gelation time are correlated.

## 2. Experiments, Methodology and Background

### 2.1. Materials

The curing process of unsaturated polyester resin (UPR) is free radical chain-growth crosslinking polymerization. In this reaction, styrene is used as a crosslinking agent to link the polyester molecules [20]. The curing starts with the opening of highly reactive initiator (peroxide) molecules, which leads to free radical formation. These radicals interact with the styrene molecules to form new radicals. Eventually, the new radicals make contact with the polyester chains and open their unsaturated C=C bonds. This leads polyester chains to be linked via styrene bridges; hence, crosslinking takes place [20].

The UPR used in this study was 40–45% styrene by weight; it is used in the industry for manufacturing large parts through vacuum-assisted resin transfer molding. Methyl ethyl ketone peroxide (MEKP) was used as an initiator. UPR and MEKP were mixed in certain ratios for 3 min to obtain the UPR-MEKP mixture to be cured. In this study, the initiator concentration was varied by using different initiator/resin weight ratios ranging from 0.5% (0.5 g initiator/100 g of UPR) up to 3%.

As the TP material used for the co-bonded samples, a LEXAN™ PC plate with a thickness of 2 mm was used. This material was chosen since it was shown to have a strong thermodynamic affinity to UPR [2].

### 2.2. Cure Kinetics (DSC) Measurements

Isothermal differential scanning calorimetry (DSC) measurements were conducted at 25 °C using a Mettler Toledo DSC to characterize the cure kinetics of the mixtures with different initiator concentrations. Mixtures had weights of about 20 mg and the DSC scans lasted for 24 h. A total of 3 specimens were tested at the initiator/resin weight ratio of 1.5%, whereas either 1 or 2 specimens were tested at the other ratios (0.5%, 1%, 2%, 2.5%, 3%). From the DSC scans, the time the DSC peak started and reached its maximum was obtained to investigate the speed of curing. In addition, to gain more insight into the cure behavior, the heat and degree of cure were calculated.

### 2.3. Gelation Time Measurements

Gelation time measurements of UPR–MEKP mixtures with different initiator/resin weight ratios were carried out utilizing an Anton Paar-Physica MCR 501 rheometer in “plate-plate” oscillatory mode. Plates had a diameter of 25 mm with a spacing of 0.5 mm between them. A strain of 1% and a frequency of 1 Hz were used. Tests were carried out isothermally at 23 °C. Storage and loss moduli were recorded, from which the gelation time was obtained as the time when the storage modulus equaled (and subsequently exceeded) the loss modulus. A total of 3 specimens were tested at initiator/resin weight ratios of 1.5%, 2%, 2.2%, and 3%; 2 specimens were tested at ratios of 1% and 2.5%.

### 2.4. Co-Bonding

**Figure 1:**
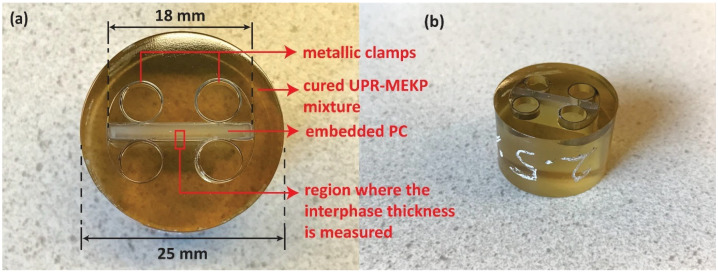
(**a**) Top and (**b**) isometric views of the co-bonded PC and UPR-MEKP.

The co-bonding of TP and TS polymers was carried out by embedding pieces of TP plates in the TS resin in cylindrical cups 25 mm in diameter. Initially, pieces of PC plates with dimensions of 18 mm × 18 mm were cut making use of a paper cutter. Later, each of the TP plate pieces was placed in the middle of the cylindrical cups with the help of metallic holders, as shown in Figure 1. Finally, the resin-initiator (UPR-MEKP) mixtures with various initiator/resin weight ratios were poured on the TP pieces up to a height of about 20 mm. The TP pieces embedded in resin were left for curing at room temperature for 24 h. At least three specimens were prepared per initiator/resin weight ratio (at 1%, 1.5%, 2%, 2.5%, 3%), except at 0.5%, where two specimens were prepared.

### 2.5. Interphase Thickness Measurements

The interphase thickness was measured using a Keyence VHX-7000 digital optical microscope equipped with a VH-100UR lens. Before microscopy, the co-bonded samples were polished using a Struers Tegramin 30 polisher. The polishing procedure involved grinding with SiC paper (500, 1000, 2000, and 4000 grits, respectively) and polishing later using several different polishing clothes with diamond solutions. Final polishing was obtained using an MD-Chem cloth with a colloidal silica suspension. During the measurements, the interphase thickness was obtained from the middle point of the cross-section, as marked in Figure 1, where the interphase thickness reached a maximum (as it was away from the metallic clamps that prevented interdiffusion).

### 2.6. Diffusivity of UPR into Thermoplastic Polymers

The diffusivity of the UPR-MEKP mixture into PC was estimated to evaluate the diffusion kinetics at different initiator initiator/resin weight ratios. It was shown by Zanjani, J.S.M., Baran, I. and Akkerman, R. [2] that the diffusion of UPR into PC is Fickian. Fick’s second law of diffusion, which was used to model the diffusion kinetics, is as follows:

(1) $$\frac{\partial C}{\partial t}=-D\frac{\partial^{2}C}{\partial x^{2}},$$

where *C* is the concentration of the diffusing species and *D* is the diffusivity (diffusion coefficient) [21]. The diffusivity *D* is a proportionality factor between the mass flux and the concentration gradient [21]. In other words, the larger the *D* is, the larger the mass flux is, given a certain concentration gradient. Assuming that *D* is constant, *C* at a certain time and location can be solved when *D* is known and the boundary conditions are input [4,21]. In our case, the interphase thickness (diffusion length) and the gelation time are known, while *D* is unknown. Hence, assuming that the interphase development stops at the gelation time [22] and using the gelation time and interphase thickness measurements, the diffusivity *D* can be calculated, as presented in detail in [4].

## 3. Results and Discussion

### 3.1. Cure Kinetics and Gelation Time

**Figure 2:**
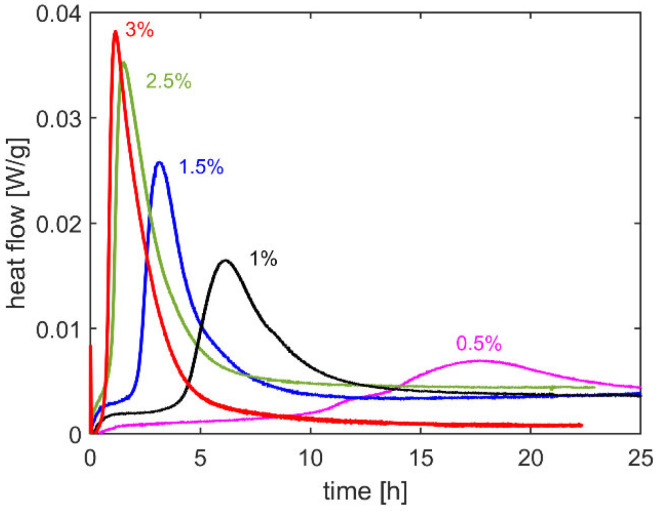
DSC curves of UPR–MEKP mixture for different MEKP/UPR weight ratios.

DSC curves of UPR–MEKP mixtures with different initiator/resin weight ratios are shown in Figure 2. All curves exhibit exothermic peaks, which correspond to the heat flow resulting from the curing reaction. According to the figure, the peaks start and reach their highest point earlier as the initiator/resin weight ratio is increased, which means that curing takes place faster. The heat of the cure is calculated as the area below the DSC peaks. For this, initially, a baseline is drawn for each DSC curve. After constructing the baselines and calculating the area between the DSC curves and the baseline, the heat of the cure is calculated, the values of which are presented in Table 1. The DSC tests carried out at the initiator/resin weight ratio of 1.5%, involving multiple specimens, had a very low scatter, showing the good repeatability of the test. Note that for the mixture with 0.5% initiator, curing was not completed (see the incomplete peak corresponding to 0.5% in Figure 2); hence, the heat of the cure was not calculated for this case. Considering the other cases, the heat of cure was seen to increase with an increase in the initiator/resin weight ratio, which is agreement with the findings of Vilas et al. [17]. This means that a higher degree of cure is reached at higher initiator/resin weight ratios, where the normalized degree of cure for different initiator/resin ratios is calculated as the ratio of the heat of cure at a certain concentration to that at the concentration of 3% (Table 1). While the initiator/resin weight ratio of 1% led to a significantly low degree of cure (0.58), for higher ratios high degrees of cure above 0.90 were obtained for all cases.

**Figure 3:**
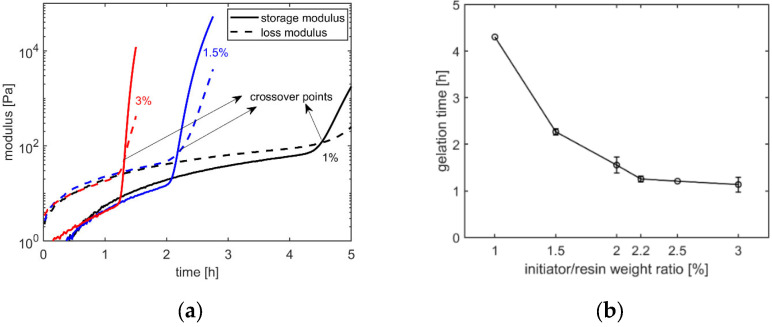
(**a**) The evolution of storage and loss modulus with time in rheometer tests and (**b**) gelation times of UPR-MEKP mixture for different MEKP/UPR weight ratios. Markers represent mean values and error bars represent ± one standard deviation (no error bar at 1% and 2.5%, since only 2 specimens were tested at these ratios).

The acceleration of curing was also confirmed by the gelation times measured in rheometer tests at a wide range of initiator/resin weight ratios. Figure 3a presents the evolution of storage and loss modulus with time from these measurements. It can be observed that the crossover time of the storage and loss modulus, which is a commonly used indicator of gelation time, is lower at a higher initiator/resin weight ratio. The gelation times taken from these measurements are plotted against the initiator/resin weight ratio in Figure 3b. It can be seen that the gelation time decreases with an increasing initiator/resin weight ratio, with the decrease being more dramatic at lower ratios. This is in agreement with the previous observations made by Kuppusamy and Neogi [15]. To illustrate, according to Figure 3b, the gelation time decreases by 47% (from 4.30 h to 2.27 h) in the initiator/resin weight ratio range of 1–1.5%, while the decrease is only 6% (from 1.21 h to 1.14 h) in the initiator/resin ratio range of 2.5–3%.

### 3.2. Interphase Morphology

**Figure 4:**
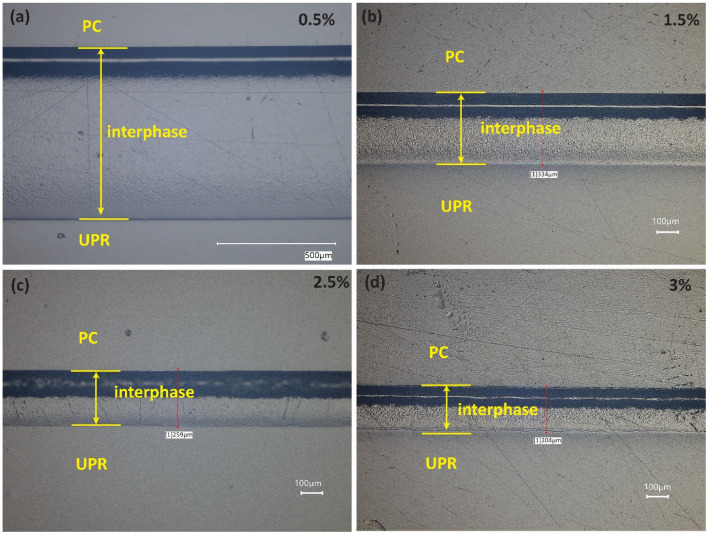
Optical micrographs of UPR–MEKP mixture for MEKP/UPR weight ratios of (**a**) 0.5%, (**b**) 1.5%, (**c**) 2.5%, and (**d**) 3%.

**Figure 5:**
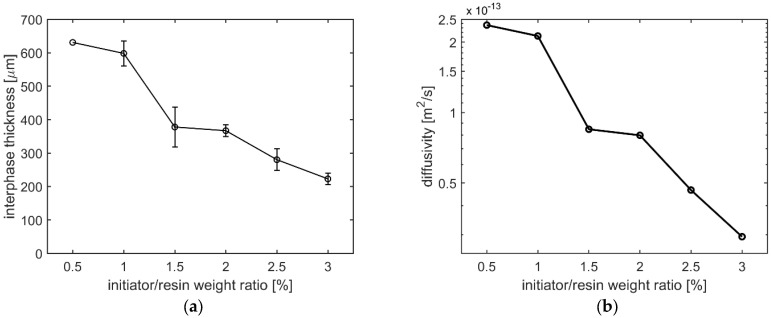
(**a**) Interphase thickness vs. MEKP/UPR weight ratio. Markers represent mean values and error bars represent ± one standard deviation (no error bar at 0.5%, since only 2 specimens were tested at that ratio). (**b**) Diffusivity vs. initiator/resin weight ratio calculated based on Equation (1) using the gelation times in Figure 3b and interphase thicknesses in Figure 5a.

The interphase between the co-bonded PC and UPR-MEKP mixtures with initiator/resin weight ratios of 0.5%, 1.5%, 2.5%, and 3% can be seen in the optical micrographs shown in Figure 4. In all micrographs, it can be seen that the interphase comprises two regions, one with a dark color and the other one with a lighter color and a pit-like morphology. When the dark region is focused during microscopy analysis, a single phase is observed, signifying that the mixing is homogeneous. On the other hand, the other region exhibits a nodular, phase-separated morphology resulting from the limited solubility of PC in the UPR–MEKP mixture [2]. The interphase thicknesses measured from the optical micrographs are plotted against the initiator/resin weight ratio in Figure 5a. Figure 4 and Figure 5a both demonstrate that the interphase thickness decreases nonlinearly with the initiator/resin weight ratio.

Interphase thickness is known to be influenced by the physical state of the TP (for instance, its temperature with respect to the glass transition temperature), the temperature-dependent viscosity, and the gelation time of the resin [2,4,10,19]. In this study, since the temperature and the TP material used were the same for experiments with different initiator/resin weight ratios, the first two factors were not effective in the observed interphase thickness vs. initiator ratio trend. Nevertheless, as also shown in Figure 3b, gelation time was highly reduced with an increasing initiator/resin weight ratios, which is considered to be the main reason for the decrease in interphase thickness. A decrease in gelation time allows less time for interdiffusion to take place, eventually leading to a lower interphase thickness [2]. In Figure 5a, the steepest decrease in the interphase thickness (by 37%, from 598 µm to 378 µm) can be observed in the initiator/resin weight ratio range of 1–1.5%, which corresponds to the range where the steepest decrease in gelation time took place in Figure 3b. The peak times of the DSC curves shown in Figure 2, which show a similar trend to the gelation times shown in Figure 3b, also agree with the trend of interphase thickness change with the initiator/resin ratio shown in Figure 5a. Although the degree of cure is low (0.58) for the mixture with initiator/resin weight ratio of 1% (Table 1), this is not thought to have contributed to the high interphase thickness via a lower viscosity of the mixture. This is because the interphase thickness evolution ceases at gelation [22,23], which takes place at a far lower degree of cure (0.15 [1] for the material system studied; it is assumed to be constant for different cure conditions based on [24,25]). Furthermore, at gelation, the complex viscosities of the resin–initiator mixtures with different initiator/resin weight ratios measured by the rheometer were in the range of 10–100 Pa·s for all initiator ratios, which is a small range considering the fast increase of viscosity at gelation. In future work, in situ interphase development should be studied for resin-initiator mixtures with different initiator/resin weight ratios to verify the cessation of interdiffusion for the material studied in this work.

Using the gelation times shown in Figure 3b and the interphase thicknesses shown in Figure 5a, the diffusivity of the resin into PC was calculated based on Equation (1), which is shown in Figure 5b. It can be seen that the diffusivity also decreases with an increase in the initiator/resin weight ratio, similar to the interphase thickness. In fact, the trends of diffusivity vs. initiator/resin weight ratio (Figure 5b) and interphase thickness vs. initiator/resin weight ratio (Figure 5a) can be seen to be quite similar. A decreasing diffusivity with an increasing initiator/resin weight ratio shows that higher ratios are less favorable for the diffusion of resin.

## 4. Conclusions

In this study, the effect of the initiator (MEKP) concentration of the resin-initiator mixture (initiator/resin weight ratio) on the interphase thickness of the co-bonded TP-TS (UPR-PC) was investigated for the first time. Gelation time was found to decrease with an increasing initiator concentration. Co-bonded polymers with higher initiator concentrations showed lower interphase thicknesses, which correlated well with the decrease seen in the gelation time. Investigating the effect of initiator concentration on the diffusion kinetics, the diffusivity of the resin was also seen to decrease with an increasing initiator concentration, showing a similar trend to that of the interphase thickness. Varying the initiator concentration helped us to exclusively investigate the effect of gelation time on the interphase thickness.

In future work, we recommend studying other TPs to check if the trend of decreasing interphase thickness with an increase in initiator concentration can be observed as well. Considering that the physical state of the thermoplastics and their affinity toward the thermoset resin both play a significant role in the interphase formation [2,8,9], investigating thermoplastics with different affinities and physical states is recommended. Furthermore, the method used for controlling the interphase thickness by varying the initiator concentration paves the way for investigating the effect of interphase thickness on the processing-induced deformations of the co-bonded TP-TS composites [3].

## Figures and Tables

**Table 1:** Heat of cure calculated from the DSC curves and the degree of cure normalized with respect to the heat of cure of mixture with 3% initiator (table shows mean values. Only for 1.5%, ± one standard deviation is also given, as 3 specimens were tested at that ratio).

Initiator/Resin Weight Ratio	Heat of Cure [J/g]	Normalized Degree of Cure [-]
1%	149.7	0.58
1.5%	233.4 ± 2.9	0.90 ± 0.01
2.5%	249.1	0.96
3%	259.9	1

## Data Availability

The data presented in this study are available on request from the corresponding author.
